# Prediction of lung papillary adenocarcinoma-specific survival using ensemble machine learning models

**DOI:** 10.1038/s41598-023-40779-1

**Published:** 2023-09-08

**Authors:** Kaide Xia, Dinghua Chen, Shuai Jin, Xinglin Yi, Li Luo

**Affiliations:** 1 Guiyang Maternal and Child Health Care Hospital, Guiyang Children’s Hospital, Guiyang, China; 2 Department of General Surgery, The Forth People’s Hospital of Guiyang, Guiyang, China; 3 School of Big Health, Guizhou Medical University, Guiyang, China; 4 Department of Respiratory Medicine, Third Military Medical University, Chongqing, China; 5 Department of Clinical Laboratory, The Second People’s Hospital of Guiyang, Guiyang, China

**Keywords:** Oncology, Risk factors, Mathematics and computing

## Abstract

Accurate prognostic prediction is crucial for treatment decision-making in lung papillary adenocarcinoma (LPADC). The aim of this study was to predict cancer-specific survival in LPADC using ensemble machine learning and classical Cox regression models. Moreover, models were evaluated to provide recommendations based on quantitative data for personalized treatment of LPADC. Data of patients diagnosed with LPADC (2004–2018) were extracted from the Surveillance, Epidemiology, and End Results database. The set of samples was randomly divided into the training and validation sets at a ratio of 7:3. Three ensemble models were selected, namely gradient boosting survival (GBS), random survival forest (RSF), and extra survival trees (EST). In addition, Cox proportional hazards (CoxPH) regression was used to construct the prognostic models. The Harrell’s concordance index (C-index), integrated Brier score (IBS), and area under the time-dependent receiver operating characteristic curve (time-dependent AUC) were used to evaluate the performance of the predictive models. A user-friendly web access panel was provided to easily evaluate the model for the prediction of survival and treatment recommendations. A total of 3615 patients were randomly divided into the training and validation cohorts (n = 2530 and 1085, respectively). The extra survival trees, RSF, GBS, and CoxPH models showed good discriminative ability and calibration in both the training and validation cohorts (mean of time-dependent AUC: > 0.84 and > 0.82; C-index: > 0.79 and > 0.77; IBS: < 0.16 and < 0.17, respectively). The RSF and GBS models were more consistent than the CoxPH model in predicting long-term survival. We implemented the developed models as web applications for deployment into clinical practice (accessible through https://shinyshine-820-lpaprediction-model-z3ubbu.streamlit.app/). All four prognostic models showed good discriminative ability and calibration. The RSF and GBS models exhibited the highest effectiveness among all models in predicting the long-term cancer-specific survival of patients with LPADC. This approach may facilitate the development of personalized treatment plans and prediction of prognosis for LPADC.

## Introduction

Lung cancer remains the leading cause of cancer-related death worldwide, accounting for approximately 1.8 million deaths^1^. In the United States of America, the 5-year survival rate of patients with lung cancer is approximately 20%^2^. Adenocarcinoma is the major histological subtype of non-small cell lung cancer^3, 4^. Recent advances in research have facilitated the classification of primary lung cancer^5^. Based on semi-quantitative assessment, the World Health Organization classified the histomorphologic growth pattern of invasive non-mucinous adenocarcinoma into five subtypes (i.e., lepidic, acinar, papillary, micropapillary, and solid)^6^. In particular, primary lung papillary adenocarcinoma (LPADC) is a rare subtype, accounting for approximately 0.84% of all lung cancer cases^7^. This subtype may originate from glandular follicular cells and often exhibits a prominent inflammatory stromal response^8^. In the early stages of LPADC, patients do not develop clinical symptoms (e.g., cough, phlegm, and fever), and are not effective in antibiotic treatment for pneumonia. Studies have investigated differences in the prognosis of different subtypes of LPADC, the evidence highlighted the importance of prognostic prediction in lung adenocarcinoma (a subtype of lung cancer with independent presentation)^9, 10^.

Due to the rarity of LPADC, most currently available studies are case reports or single-center small-sample investigations. The 5-year overall survival rate of LPADC patients is less than 35%, and Cox proportional hazards regression models constructing nomograms based on tumor characteristics, demographic characteristics, and treatment modalities are the traditional methods used to predict survival in LPADC^11^. Previous studies have also explored the use of machine learning algorithms in the diagnosis and prognosis of small cell lung cancer in the lung^12–14^.Of note, Cox models often rely on the restrictive assumption of proportional risk. In addition, when using this approach, it is important to consider whether the association between predictors and hazards is suitable for modeling, and whether nonlinear effects or higher-order interactions of predictors should be included^15, 16^. To overcome this limitation, the evolution of machine learning provides an alternative to semi-parametric modeling by relaxing the assumptions of the data generation mechanism and taking into account all possible interactions between variables and influence correction^17^.

Few studies have used integrated machine learning algorithms to assess the prognosis of patients with lung adenocarcinoma, even fewer studies have used the output of predictive models to aid clinical practice^18^. Therefore, this study used a sample of patients with LPADC from the Surveillance, Epidemiology and End Results (SEER) database to develop and validate an integrated machine learning model for the prediction of LPADC cancer-specific survival (CSS). The objectives were to support clinical decision-making in LPADC, and develop a web-based calculator for estimating the individual probability of CSS for patients with lung adenocarcinoma. The selection of studies was based on the TRIPOD report checklist^19^.

## Materials and methods

### Patient selection

**Figure 1:**
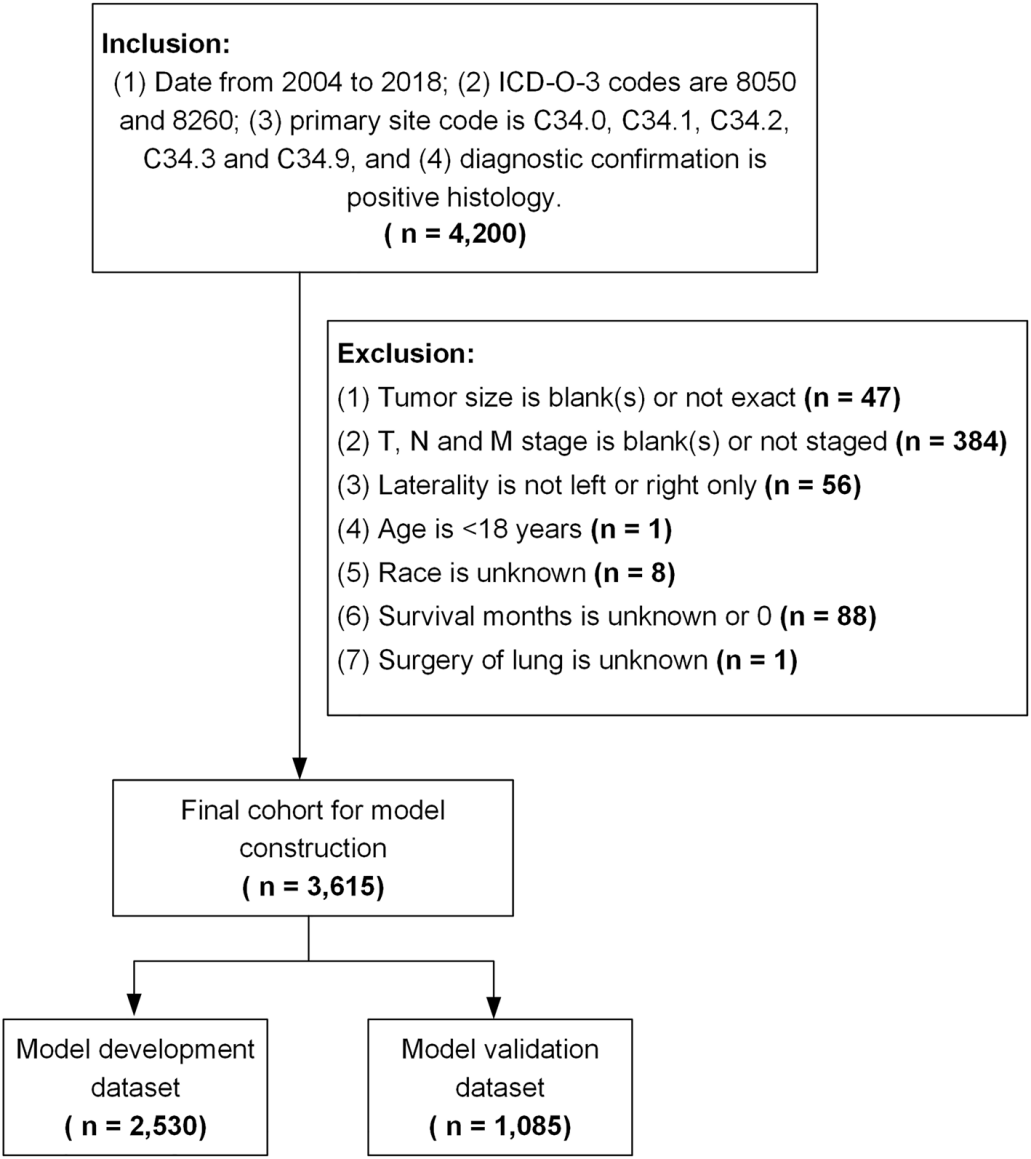
Screening process for the selection of patients. ICD-O-3, International Classification of Diseases for Oncology (Third Edition).

The SEER*Stat version 8.4.0 (https://seer.cancer.gov/seerstat/) software was used to select patients with LPADC from the version of the SEER research plus database (18 registries, with additional treatment fields, 2000–2018) based on November 2019 submissions. The inclusion criteria were as follows: (I) diagnosis from 2004 to 2018; (II) International Classification of Diseases for Oncology, Third Edition, histologic type codes 8260 and 8050; (III) primary site codes C34.0–C34.9; and (IV) diagnostic confirmation through histology. The exclusion criteria were as follows: (I) blank or not exact tumor size; (III) unknown tumor-node-metastasis (TNM) stage; (IV) tumor laterality in both lungs; (V) age < 18 years; and (VI) unknown race, survival months, and surgery status (Fig. 1). The SEER database is publicly accessible; hence, there was no requirement for additional ethical approval.

### Cohort definition and variables

We randomly classified the study sample into the training and validation cohorts using a 7:3 ratio. The training and validation cohorts were used to construct and verify the model, respectively. Fourteen variables from the SEER database were included in the study model, including demographic variables (age at diagnosis, sex, race, and marital status), tumor characteristics (laterality, TNM stage, grade, tumor size, and primary site), and treatment status (chemotherapy, surgery, and radiotherapy). Based on the age at diagnosis and tumor size, X-tile software (https://medicine.yale.edu/lab/rimm/research/software/) was used to determine the optimal cut-off values for category-based conversion of the measures and also to maximize the difference between categories after conversion^20, 21^. The marital status was either married or other, while the cancer grade was I–II, III–IV, or unknown. Primary sites in the lung were classified as lower, middle, upper, other, and not otherwise specified. The three surgical approaches to the primary site were no surgery, lobectomy, and other surgery. The dummy variable design for disordered multicategorical variables was performed using the ‘get_dummies’ function in the pandas package. In the present study, the eighth edition of TNM staging was used after manual conversion coding. CSS was defined as death specifically due to LPADC and used as the outcome variable of interest in this study.

### Model development

Categorical variables were collated in frequency and percentage format, and differences between groups were compared using the χ^2^ test. Four prognostic models, including three ensemble learning models (i.e., gradient boosting survival [GBS] analysis, random survival forest [RSF], and extra survival trees [EST]) and a Cox proportional hazards regression (CoxPH) model, were used to analyze the CSS rates of patients with LPADC. The area under the time-dependent receiver operating characteristic curve (time-dependent AUC) and Harrell’s concordance index (C-index) were used to evaluate the discriminative ability of these models^22^. Evaluation of the calibration capability of the prediction model was performed using the integrated Brier score (IBS). Furthermore, we visualized feature importance (‘PermutationImportance’ function) in the models using the training dataset. A web-based calculator for the probability of CSS in patients with LPADC was deployed, presenting the estimated prognostic survival curves and 3-, 5-, and 10-year survival rates. All machine learning models, statistical analysis, and visualization were implemented in Python version 3.9 (Python Software Foundation for Statistical Computing, Wilmington, DE, USA) using the scikit-survival^23^, tableone^24^, and eli5 packages.

### Ethics statement

The SEER database is free for researchers to download and therefore does not require ethical review by the authors’ institution.

## Results

### Patient characteristics

The best cutoff values for age and tumor size were 79 years and 28 and 52 mm, respectively. Age was divided into two age groups (i.e., < 79 and ≥ 79 years), while tumor size was divided into four groups (i.e., < 28, 28–52, > 52 mm, and unknown). A total of 3,615 patients diagnosed with LPADC (2004–2018) were included in this analysis. After randomization, there were 2,530 and 1,085 patients in the training and validation cohorts, respectively. Overall, 86% of the patients were younger than 80 years; the sample included a slightly higher number of females (51.6%) than males (48.4%). LPADC was more likely to occur on the right side (58.6%) of the lung; 67% of patients had pre-T3 stage disease without regional lymphatic metastases. 23% of patients had distant metastases, while 60% had low-grade disease and tumor size < 28 mm, mostly in the lower and upper parts of the lung (86%). Moreover, 80% and 65% of the patients did not receive radiotherapy and chemotherapy, respectively. Lobectomy was performed in more than half of the patients. Other surgical procedures were performed in 18% of the patients, while nearly 30% of the patients did not undergo surgery. Based on the χ^2^ test, there was no difference in the correlation index between the two cohorts generated by the random split, indicating that these groups were comparable (Table 1).

**Table 1:** Clinical, pathological, and treatment characteristics of patients with lung papillary adenocarcinoma (LPADC).

Characteristics	Overall	Training	Validation	p-value
	n (%)	n (%)	n (%)	
Age, years				
< 79	3098 (85.7)	2161 (85.4)	937 (86.4)	0.489
≥ 79	517 (14.3)	369 (14.6)	148 (13.6)	
Sex				
Female	1865 (51.6)	1304 (51.5)	561 (51.7)	0.957
Male	1750 (48.4)	1226 (48.5)	524 (48.3)	
Race				
Black	376 (10.4)	264 (10.4)	112 (10.3)	0.481
Other	408 (11.3)	275 (10.9)	133 (12.3)	
White	2831 (78.3)	1991 (78.7)	840 (77.4)	
Laterality				
Left	1496 (41.4)	1055 (41.7)	441 (40.6)	0.580
Right	2119 (58.6)	1475 (58.3)	644 (59.4)	
T stage				
T1	1445 (40.0)	979 (38.7)	466 (42.9)	0.069
T2	1005 (27.8)	716 (28.3)	289 (26.6)	
T3	540 (14.9)	396 (15.7)	144 (13.3)	
T4	625 (17.3)	439 (17.4)	186 (17.1)	
N stage				
N0	2413 (66.7)	1665 (65.8)	748 (68.9)	0.340
N1	337 (9.3)	243 (9.6)	94 (8.7)	
N2	655 (18.1)	471 (18.6)	184 (17.0)	
N3	210 (5.8)	151 (6.0)	59 (5.4)	
M stage				
M0	2781 (76.9)	1927 (76.2)	854 (78.7)	0.105
M1	834 (23.1)	603 (23.8)	231 (21.3)	
Marital status				
Married	2037 (56.3)	1402 (55.4)	635 (58.5)	0.091
Other	1578 (43.7)	1128 (44.6)	450 (41.5)	
Grade				
I–II	2139 (59.2)	1490 (58.9)	649 (59.8)	0.424
III–IV	329 (9.1)	223 (8.8)	106 (9.8)	
Unknown	1147 (31.7)	817 (32.3)	330 (30.4)	
Tumor size, mm				
28–52	1173 (32.4)	833 (32.9)	340 (31.3)	0.413
< 28	1776 (49.1)	1225 (48.4)	551 (50.8)	
> 52	471 (13.0)	328 (13.0)	143 (13.2)	
Unknown	195 (5.4)	144 (5.7)	51 (4.7)	
Primary site				
Lung NOS	167 (4.6)	124 (4.9)	43 (4.0)	0.380
Lower	1442 (39.9)	985 (38.9)	457 (42.1)	
Middle	265 (7.3)	190 (7.5)	75 (6.9)	
Other	77 (2.1)	55 (2.2)	22 (2.0)	
Upper	1664 (46.0)	1176 (46.5)	488 (45.0)	
Chemotherapy				
No/Unknown	2356 (65.2)	1642 (64.9)	714 (65.8)	0.627
Yes	1259 (34.8)	888 (35.1)	371 (34.2)	
Surgery group				
Lobectomy	1941 (53.7)	1351 (53.4)	590 (54.4)	0.557
No surgery	1040 (28.8)	741 (29.3)	299 (27.6)	
Other surgery	634 (17.5)	438 (17.3)	196 (18.1)	
Radiation				
No/Unknown	2890 (79.9)	2027 (80.1)	863 (79.5)	0.724
Yes	725 (20.1)	503 (19.9)	222 (20.5)	

*NOS* not otherwise specified.

### Model application and performance

**Figure 2:**
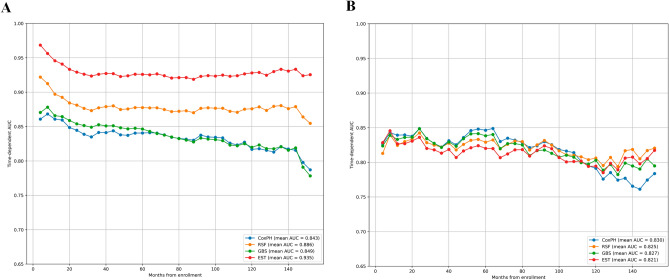
Time-dependent receiver operating characteristic curve for the training (**A**) and validation (**B**) cohorts.

To ensure comparability, we used all the features for the construction and validation of the models. In the training cohort, the EST model had the largest time-dependent AUC, followed by the RSF, CoxPH, and GBS models. The mean time-dependent AUC for the EST, RSF, CoxPH, and GBS models were 0.935, 0.886, 0.843, and 0.849, respectively. In the training cohort, the time-dependent AUC showed that the GBS and CoxPH models progressively abolished their discriminative ability for the prediction of long-term survival (Fig. 2A). In the validation cohort, the discriminative ability of the four prediction models tended to be similar. According to the time-dependent AUC, the EST and RSF models did not exhibit a similar performance to that observed in the training cohort. The highest mean value of the time-dependent AUC was 0.821, 0.825, 0.830, and 0.827 for the EST, RSF, CoxPH, and GBS models, respectively; according to these findings, the EST model exhibited the worst performance. In terms of time trends, the RSF model and GBS performed more consistently across time than the other models, while the CoxPH model performed less well for long-term forecasts after 10 years (Fig. 2B).

The C-index analysis yielded similar findings to those noted with the time-dependent AUC. In the training cohort, the EST model exhibited the best performance (C-index: 0.850), followed by the RSF, GBS, and CoxPH models; the IBS also showed similar results. In the validation cohort, the CoxPH model had the largest C-index value (0.783), followed by the GBS, RSF, and EST models. In the validation cohort, the RSF and GBS models had the lowest IBS (0.16), whereas the EST model had the highest IBS (0.166) (Table 2).

**Table 2:** Performance of the models.

Model	Training cohort		Validation cohort	
	C-index	IBS	C-index	IBS
CoxPH	0.798	0.156	0.783	0.162
RSF	0.816	0.137	0.776	0.160
GBS	0.807	0.153	0.780	0.160
EST	0.850	0.110	0.773	0.166

*CoxPH* Cox proportional hazards, *EST* extra survival trees, *GBS* gradient boosting survival, *IBS* integrated Brier score, *RSF* random survival forest.

### Feature importance

**Figure 3:**
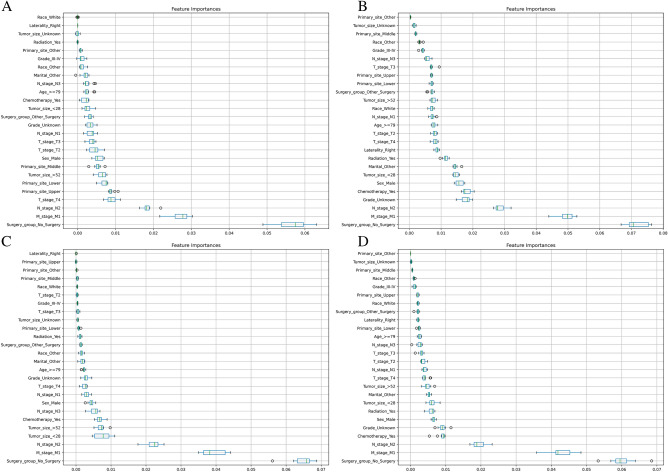
Feature importance plot of the CoxPH (**A**), EST (**B**), GBS (**C**), and RSF (**D**) models. *CoxPH* Cox proportional hazards, *EST* extra survival trees, *GBS* gradient boosting survival, *RSF* random survival forest.

The feature importance plot shows the contribution of each feature in the prognostic model. N2 stage, M1 stage, and no surgery occupied the top three positions in the feature importance ranking; this ranking was consistently observed across the four models. In the CoxPH model, T4 stage, and tumor primary location (lower and upper) were more important than other features. In the machine learning survival model, the most important features were chemotherapy, tumor size, grade unknown, and sex (Fig. 3).

### Algorithm deployment

**Figure 4:**
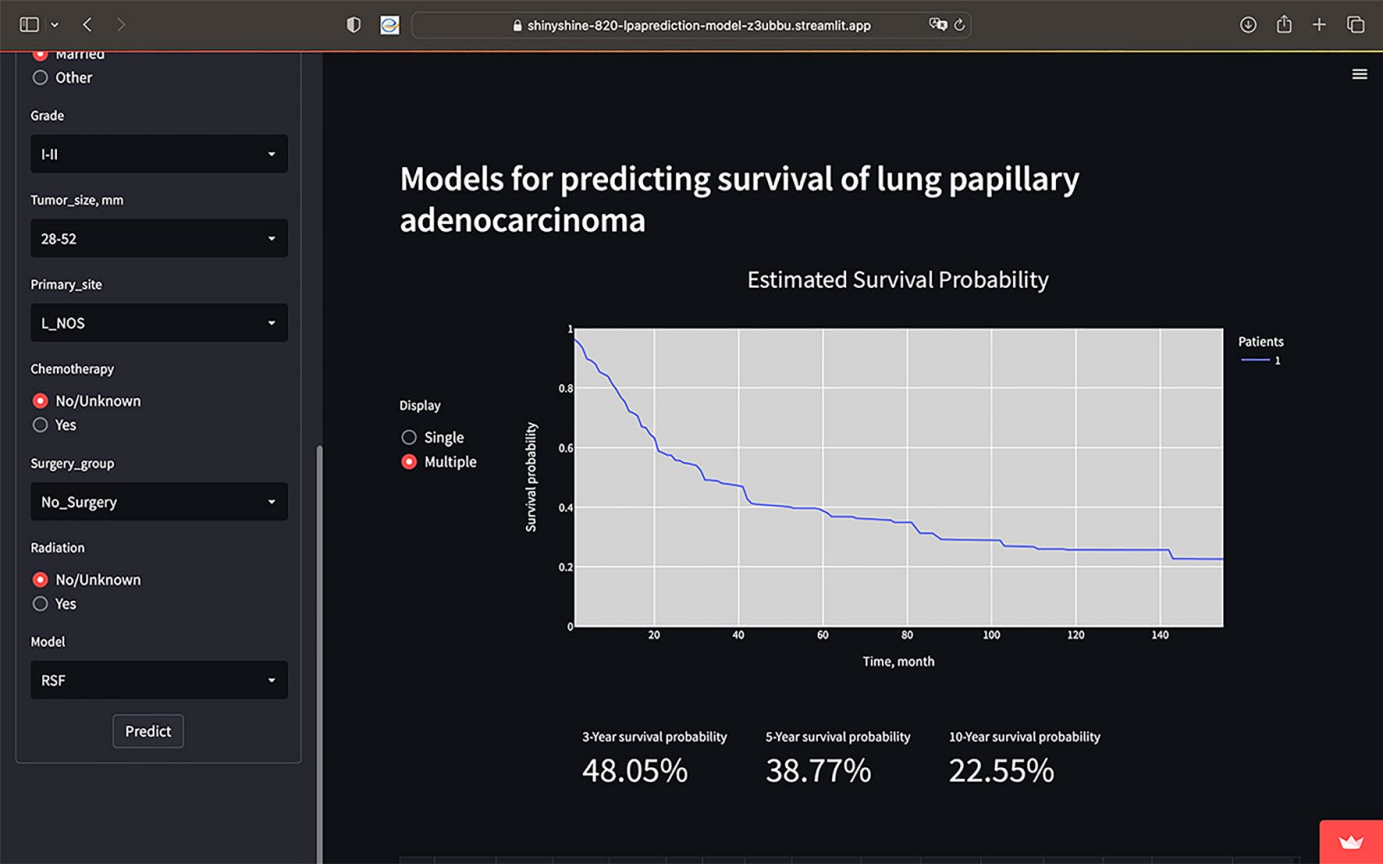
Interface display of web calculator. A patient with LPADC aged < 79, male, black, left-sided lung, T1N0M0 stage, married, grade I-II, tumor size of 28–52 mm, primary site of lung (NOS), and no radiotherapy, chemotherapy or surgery was performed. His CSS at 3-, 5- and 10- year were 48.05%, 38.77% and 22.55%. *LPADC* lung papillary adenocarcinoma, *NOS* not otherwise specified, *CSS* cancer-specific survival.

The constructed models for determining the CSS rate of patients with LPADC were deployed on a web page. The functionality of the application and the visualization of the output are shown in the following Fig. 4. The web application, primarily used for research or informational purposes, can be publicly accessed at https://shinyshine-820-lpaprediction-model-z3ubbu.streamlit.app/.

## Discussion

The accurate prediction of survival in patients with LPADC is essential for patient counseling, follow-up, and treatment planning. Previous studies have revealed multiple prognostic factors that affect the survival time of patients with pulmonary papillary carcinoma, including patient age, grade classification, lymph node status, tumor size, distant metastases, and surgical treatment^9, 11^. Machine learning is increasingly utilized in research for the prediction of survival of patients with cancer^25–27^, with relatively favorable results. Although CoxPH is the classical method utilized for the analysis of survival data, the use of this method requires linear relationships between variables. As a result of the continuous advances achieved in recent years, machine learning is widely applied to the medical field^28–30^. In this study, we used ensemble machine learning models to accurately predict CSS in patients with LPADC, and obtained satisfactory results.

Consistent with the findings reported by You et al., the four models developed in this study confirmed that surgery is an important prognostic factor for patients with lung adenocarcinoma^3^. Similarly, distant metastases have an important impact on the prognosis of patients with LPADC. In conjunction with previous analyses, the findings demonstrate that patients who developed distant metastases had poorer survival rates than other patients^26, 27^. A higher N-stage also plays a crucial role in the model, indicating poor prognosis^28^. Other characteristics (e.g., tumor size, grade, sex, chemotherapy, primary site, etc.) have different degrees of importance in various models^11, 23, 27^. These results suggest that the selection of appropriate treatment modalities (e.g., surgery, radiotherapy, and chemotherapy) may be more important for predicting CSS in patients with LPADC than TNM staging alone.

Interestingly, the ensemble models (i.e., GBS, EST, and RSF) did not demonstrate a markedly better ability for predicting CSS in LPADC in the validation cohort compared with the CoxPH model. This indicates that the machine learning approach may only offer advantages when traditional models are limited. Therefore, there are several possible explanations for the comparable predictive performance observed between the ensemble and CoxPH models in this study. Firstly, the number of predictors used to construct the model was not sufficiently large, and the advantages of machine learning in analyzing large samples and multivariate data are not fully realized. Secondly, the SEER database collects variables derived from clinical experience; many of these variables are linearly correlated with outcomes. Therefore, the data may be better qualified for the application of parametric (CoxPH) models. The GBS, EST, and RSF models developed in this study achieved the predictive efficacy of the CoxPH model under a broader condition. The web calculator constructed for the study is based on the training dataset, and care should be taken when applying the EST model that may be overconfident. Hence, it is not recommended to use this algorithm for the prediction of survival. In this study, the CoxPH model had poorer long-term predictive power than the ensemble models. Therefore, use of the RSF model is recommended for the prediction of LPADC CSS beyond 10 years.

This study had several limitations. Firstly, in the SEER database, there was a lack of data regarding established predictors of survival in patients with LPADC (e.g., chemotherapy regimens and biological markers). Secondly, due to the retrospective nature of this study and data processing, samples with missing information were excluded; this may have led to considerable bias. Thirdly, the work related to the measurement of prediction model errors in the study is not yet complete. Finally, the results of this study were not externally validated; although we randomly split the study sample during the development of the models, the generalizability and reliability of this approach should be further validated with external datasets. The prognostic value of this approach should be improved in the future by adding more predictors, increasing external validation, and conducting prospective studies.

In conclusion, a geometric model and a CoxPH model were developed and evaluated for the prediction of CSS in patients with LPADC. Overall, all four models showed excellent discriminative and calibration capabilities; in particular, the RSF model and GBS model showed excellent consistency for long-term forecasting. The integrated web-based calculator offers the possibility to easily calculate the CSS of patients with LPADC, providing clinicians with a user-friendly risk stratification tool.

### Supplementary Information

Supplementary Information.

## Data Availability

The original contributions presented in the study are included in the article, further inquiries can be download from https://github.com/ShinyShine-820/LPAprediction.
